# The effectiveness of physiotherapy treatment on balance dysfunction and postural instability in persons with Parkinson’s disease: a systematic review and meta-analysis

**DOI:** 10.1186/s13102-016-0042-0

**Published:** 2016-06-06

**Authors:** Asmare Yitayeh, Amare Teshome

**Affiliations:** Department of Physiotherapy, School of Medicine, College of Medicine and Health Sciences, University of Gondar, Gondar, Ethiopia; Department of Dentistry, School of Medicine, College of Medicine and Health Sciences, University of Gondar, Gondar, Ethiopia

**Keywords:** Randomized controlled trials, Parkinson’s disease, Physiotherapy, Postural instability, Balance dysfunction, Exercise, Equilibrium, Postural control, Rehabilitation

## Abstract

**Background:**

Balance dysfunction and postural instability in Parkinson’s disease are among the most relevant determinants of an impaired quality of life. Physiotherapy interventions are essential to reduce the level of disability by treating balance dysfunction and postural instability. The aim of this systematic review with meta-analysis was to test the effectiveness of conventional physiotherapy interventions in the management of balance dysfunction and postural instability in Persons with idiopathic Parkinson’s disease.

**Method:**

A systematic literature search of the Cochrane Library, PubMed/Medline, PEDro, Rehadat, and Rehab Trials were performed by 2 reviewers (AY and AT) independently. Eligible randomised controlled trials published from September 2005 to June 2015 were included. The selected RCTs, which investigated the effects of conventional physiotherapy treatments in the management of postural instability and balance dysfunction in Persons with Parkinson’s disease, were assessed on a methodological quality rating scale. Included studies differed clearly from each other with regard to patient characteristics, intervention protocol, and outcome measures. Important characteristics and outcomes were extracted, summarized and analyzed.

**Results:**

Eight trials with a total of 483 participants were eligible for inclusion of which 5 trials provide data for meta-analysis. Benefits from conventional physiotherapy treatment were reported for all of the outcomes assessed. The pooled estimates of effects showed significantly improved berg balance scale (SMD, 0.23; 95 % CI, 0.10–0.36; *P* < 0.001) after exercise therapy, in comparison with no exercise or sham treatment. Exercise interventions specifically addressing components of balance dysfunction demonstrated the largest efficacy with moderate effect size (SMD, 5.98; 95 % CI, 2.29–9.66; *P* < 0.001). Little effects were observed for interventions that specifically targeted Falls efficacy scale. The pooled data indicated that physiotherapy exercises decreased the incidence of falling by 6.73 (95 % CI: −14.00, 0.54, *p* = 0.07) with the overall effect of *Z* = 1.81.

**Conclusion:**

Physiotherapy interventions like balance training combined with muscle strengthening, the range of movement and walking training exercise is effective in improving balance in patients with Parkinson’s disease and more effective than balance exercises alone. Highly challenging balance training and incremental speed-dependent treadmill training can also be part of a rehabilitation program for management of balance dysfunction and Postural instability in patients with idiopathic Parkinson’s disease.

## Background

Parkinson’s disease (PD) is a debilitating chronic neurodegenerative illness resulting in motor dysfunction, which leads to weakness, pain, and tightness, difficulty in walking, rising from chairs, clumsy movements and a decline in physical activity. It is the second most common neurological disease in the world that affects neurophysiologic function, movement abilities, and quality of life (QOL) [1–5].

Balance dysfunction (BD) and Postural instability (PI) are the common incapacitating symptoms of PD. Untreated BD and PI can lead to increased frequency of falls and injuries which in turn increases the chance of developing Comorbidity and disability by causing alterations in postural control strategies during standing tasks and when performing voluntary movements [5–7]. Balance dysfunction and PI are also associated with a loss of equilibrium, sudden falls, progressive loss of independence and immobility [8–10].

Balance dysfunction and PI usually occur in the middle-later stages of the disease and became a clinical concern since they are not easily amenable to treatment with medication [11, 12]. Although Patients with PD get the best available medications, they still experience a declining of body function, daily activities, participation and weakening in mobility [13].

Recently, a number of systematic reviews assessed the effect of physiotherapy treatments or exercises in the management of balance dysfunction and postural instability among patients with idiopathic PD [14–18]. Although the results seem promising,most studies included in the systematic review have a small number of patients enrolled in their included studies and methodological limitations such as limited quality and a limited set of relevant outcome measures. This makes their result inconclusive about the use of physiotherapy treatments in the management of BD and PI bias [12, 19, 20].

Therefore, this systematic review aimed to evaluate the effectiveness of conventional physiotherapy treatments in improving balance and postural stability among persons with idiopathic PD.

## Method

### Protocol and registration

The systematic review was done using the preferred reporting items for systematic reviews and Meta-analysis (PRISMA) checklist.

There was no registration done either for the protocol or the systematic review.

### Eligibility criteria

A study was included if it met the following criteria:

- Randomized controlled trial methodology (level 1b evidence according to Oxfords level of evidence criteria [21] (see Table 1).
- Quality rating of greater than or equal to 5 by PEDro score;
- The target population was individuals with idiopathic PD of any time duration;
- The effects of different conventional physiotherapy treatment techniques or exercise interventions were compared with control or comparison groups,
- The primary outcomes included at least one of the following: postural instability, deficits in balance demanding activities, or risk of falling
- The article was available in English.

**Table 1:** Hierarchies of evidence for questions of therapy, prevention, aetiology or harm [CEBM]

Level 1a:	Systematic reviews (with homogeneity) of randomized controlled trials (RCTs)
Level 1b:	Individual RCTs (with narrow confidence interval)
Level 1c:	All or none studies
Level 2a:	Systematic reviews (with homogeneity) of cohort studies
Level 2b:	Individual cohort study or low quality RCTs (e.g. <80 % follow-up)
Level 2c:	“Outcomes” Research; ecological studies
Level 3a:	Systematic review (with homogeneity) of case-control studies
Level 3b:	Individual case-control study
Level 4:	Case-series (and poor quality cohort and case-control studies)
Level 5:	Expert opinion without explicit critical appraisal, or based on physiology, bench research or ‘first principles’

A study was excluded: −If the effects of non-exercise interventions were evaluated (like behavioral interventions), If other study designs than RCT were used and If quality rating was 4 or less as determined by PEDro score.

### Data sources and search strategy

Five databases (Cochrane Library, PubMed/Medline, PEDro, Rehadat, and Rehab Trials) were used during article selection process from February 2015 to September 2015. An electronic database search for relevant Randomized controlled trials (RCTs) which examined physiotherapy techniques used to treat, BD and PI among people with PD of any duration and published in international medical journals in the English language from 2005 to June 2015was conducted. We(AY, AT) searched articles using keywords of *RCTs, Parkinson’s disease, physiotherapy, postural instability, balance dysfunction, Exercise, equilibrium, postural control, and rehabilitation.*

The relevance of the reviewed studies was checked based on their topic, objectives, and methodology. Preliminary assessments have been made and some articles were excluded at the first step just by looking at the topic. On the second step, abstracts have been seen and were excluded if they did not match to the current study objectives. For the rest, the whole content of the articles was accessed and selected based on the independent and dependent variables under review.

### Type of intervention

The intervention was chosen if the RCTs used one of the following conventional physiotherapy treatment techniques: stretching, aerobic training, relaxation and muscle activation, strengthening exercises and treadmill walking.

### Type of outcomes

The primary outcomes of this study were changes in berg balance scale and falls efficacy scale among the intervention and control group at the end of the follow-up. However, there are some other secondary outcome measures used in this systematic review with Meta-analysis (Table 2).

**Table 2:** Characteristics of included randomized controlled trials

Authors	Participant characteristics	Intervention types and intensity for experiment and control groups	Outcomes
(Ashburn et al. 2007) [7]	• *n* = 142 (Exp = 70, Control = 72).	Exp group: muscle strengthening, range of movement, balance training, walking training and Strategies for falls prevention, movement initiation and compensation.	• Rates of Falling
	• Sex :male = ___female = ___	Con group: visited by nurse For 6 months	• Functional reach
	• Mean Age of expt. =72.7(9.6)		• BBS timed up and go test
	• Mean Age of control. =71.6(8.8)		
	• Baseline UPDRS: Exp = 19.8(8.3) and Control = 22.2(11.9)		
(Smania et al. 2010) [8]	• *n* = 64 (Exp = 33, control = 31)	Exp group: Exercises of self-destabilization of the COBM, Inducing destabilization of COBM externally and coordination between leg and arm movements during walking &locomotor dexterity over an obstacle course	• BBS
	• Mean Age of expt. =67.64 (7.41)	Cont.group:- active joint mobilization, muscle stretching, and motor coordination exercises.	• ABC
	• Mean Age of control = 67.26(7.18)	21 treatment sessions of 50 min each for one month.	• UPDRS
	• idiopathic PD and PI (Hoehn and Yahr [H&Y] stage 3–4)		• modified Hoehn and Yahr scale
(Protas et al., 2005) [24]	*n* = 18(Expt. = 9, Control = 9)	Exp group I: Gait training(walking on a treadmill at a speed greater than over ground walking speed)	Gait parameters
	Mean age of exp. = 71.3(7.4)	Exp group II [PNF]: Basic and Gait PNF, movement guidance, support & resistance for 1 h/day, three times per week for 8 weeks	5-step test report of falls
	Mean age of contrl. = 73.7 (8.5)		
(Schlenstedt et al. 2015) [27]	*n* = 32(Res. Training : *n* =17, balance training: *n* = 15;	2x/week for 7 weeks, Each session lasted 60 min.	Fullerton Advanced Balance (FAB) scale
	Mean age of exp. = 75.7 ± 5.5	Resistance training group: strengthening exercise was given to lower limb muscles	Timed-up-and-go-test (TUG)
	Mean age of contl. = 75.7 ± 7.2	Balance training group : stance- and gait tasks which require feed forward and feedback postural control	UPDRS
(Conradsson et al. 2015) [25]	(*n* = 100), experimental group = 51	Expt: reactive postural adjustments to control their balance during single-tasking(a 10-week Hi Balance program)	• Mini BESTest,
	Control group =49.	Control: normal physical activities and participation in ongoing rehabilitation program.	• gait velocity
	Mean Age of expt. =72.9 (6.0)		• Falls Efficacy Scale
	Mean Age of control. =73.6 (5.3)		
(Shen and Mak 2014) [29]	*n* = 51, (Expt., = 26) and (Contrl, = 25).	Expt : technology assisted balance + gait training	• falls rate
	Mean Age of expt. =63.3 (8.0)	Control :- strengthening exercises (3 sessions/week, separated by 4 weeks of selfsupervised home-based training at a frequency of 5 sessions/week	• single-leg-stance time,
	Mean Age of control. =65.3 (8.5)		• stride length
(Allen et al. 2010) [26]	*n* = 45 (Expt. =21 and Contrl. = 24)	Exp’t: Multi component exercise program (home-based)	• falls risk score
	Mean Age of expt. =66 (10)	3 sessions/week/40-60 min/session/week for 30 days for 72 sessions	• timed sit-to-stand
	Mean Age of control. =68 (7)	Control: Usual care (no exercise)	• falls rate
(Cakit et al. 2007) [28]	*n* = 31 (expt. = 21, control = 10), mean age =71.8 ± 6.4	Experimental group: Incremental speed-dependent treadmill training for 8 weeks.	• UPDRS
	baseline UPDRS 18.14 _ 9.32	control group: not really mentioned	• BBS
			• Dynamic Gait Index
			• FES

### Data extraction and analysis

Two reviewers (AY, AT)extracted data from the selected RCT studies using pre-designed forms independently. Any conflict between these two reviewers was resolved by consensus. From the selected studies, the following parameters were extracted; demographic variables (mean age, sample size), Initial and Final results of used outcome measures, and the type of intervention given along with the duration of follow-up (Table 2).

Data which are suitable to meta-analysis were entered and analyzed using RevMan 5.3 software. The difference in percentage in each treatment was recorded. When there is no documented difference, it was calculated by extracting the mean change in the experimental and control group.

### Quality assessment

The selected RCTs were critically appraised with 11 items of PEDro scale scores extracted from the Physiotherapy Evidence Database (www.pedro.org.au), 10 of which were scored using explicit decision rules. The PEDro scale assesses the methodological quality of a study based on important criteria, such as concealed allocation, intention-to-treat analysis, and adequacy of follow-up.

These characteristics make the PEDro scale a useful tool to assess the methodological quality of physical therapy and rehabilitation trials. The PEDro scale is based on a Delphi list [22] and consists of 11 items. Items 2–9 refer to the internal validity of a paper, and items 10 and 11 refer to the statistical analysis, ensuring sufficient data to enable appropriate interpretation of the results [23].

Item 1 is related to the external validity and therefore not included in the total PEDro score Item 4 (baseline similarity) was considered to be fulfilled if there were no significance (*p* > 0.05) difference between groups at baseline for one key outcome measure. Only one outcome had to achieve baseline similarity, in the case of more than one outcome is measured by the trials to fulfill item 4 criteria. The trials were rated independently by two investigators. Studies were excluded in the subsequent analysis if the cut-off of 5 points was not reached on PEDro scale score.

The following data were extracted from the included trials: study design, subject information, and description of interventions between the control and experimental group, outcome measures, outcome data, follow-up period. These data were then compiled into a prepared table. The two reviewers who selected the appropriate studies also extracted the data and evaluated the risk of bias. Data at baseline, post-treatment and follow-ups were extracted for interested outcomes.

### Data analysis

#### Qualitative analysis

The necessary information was extracted from each original study by using a format prepared in Microsoft Excel Spreadsheet.

#### Quantitative analysis (Meta-Analysis)

Meta-analysis was performed using the Review manager (RevMan5.3) software. The post-intervention data were used to obtain the pooled estimate of the immediate effect of physiotherapy interventions and effects beyond intervention period. Heterogeneity between trials was assessed using the I^2^ statistic. Heterogeneity was considered substantial if I^2^ was greater than 50 % and a random effects model applied; otherwise, a fixed effects model was used for the analysis. The pooled data for each outcome were reported as weighted mean differences (MD) with a 95%CI.

## Results

### Search yield

**Fig. 1:**
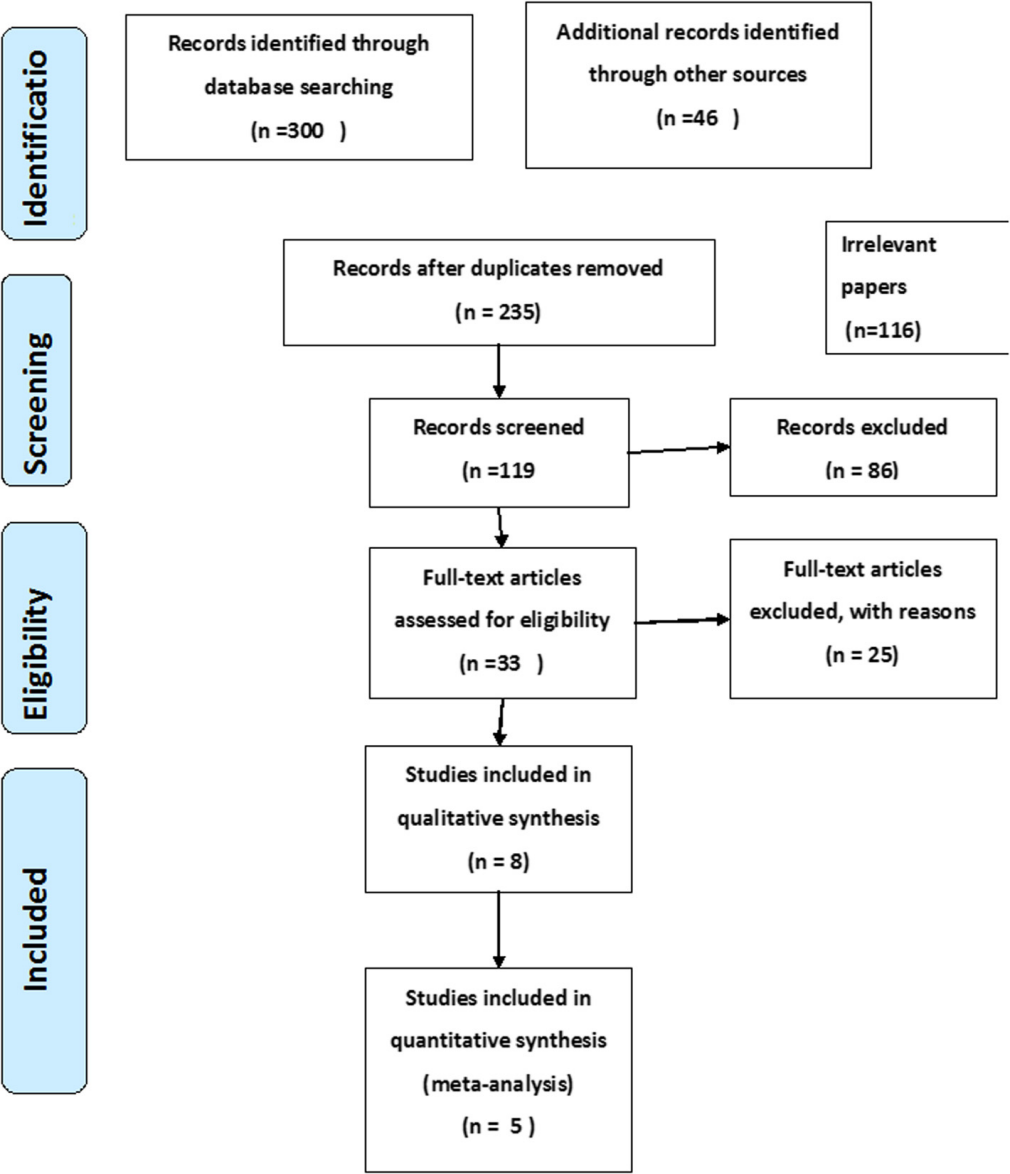
PRISMA Flow diagram showing the flow of information in the procedure of including studies in systematic review, 2015, Ethopia

A total of 346 records were identified from electronic search and additional records but 131 were duplicates. After screening title, abstracts, and references 119 papers were removed. The full-text article was obtained for 33 papers of which 25 papers were eliminated as they did not meet inclusion criteria and therefore, 8Studies included in the qualitative synthesis and 5 of them included in quantitative synthesis (see Fig. 1).

### Characteristics of included trials

All 8 trials involved a total of 483 participants and investigated the effectiveness of physiotherapy treatment and Exercise on improving postural stability and balance in Persons with Parkinson’s disease. All trials were conducted in between September 2005 and June 2015 (see Table 2).

#### Quality

The mean PEDro scores of the included trials were 7. Three studies [8, 24, 25] blinded participants, two studies [8, 24] blinded therapists and the other five trials did not, due to innate difficulties. Concealed allocations of participants were stated clearly in only two studies [25, 26] and the intention to treat analysis was considered by only three studies [25–27]. The quality assessment scores and the decisions of each item for the included trials are shown in Table 3.

**Table 3:** PEDro criteria and summary of quality assessment scores of Included studies (*n* = 8)

Criteria	(Ashburn et al., 2007) [7]	(Smania et al., 2010) [8]	(Protas et al., 2005) [24]	(Schlenstedt et al., 2015) [27]	(Conradsson et al., 2015) [25]	(Shen and Mak, 2014) [29]	(Allen et al., 2010) [26]	(Cakit et al., 2007) [28]
Eligibility criteria	✓	✓	✓	✓	✓	✓	✓	✓
Random allocation	1 Block	1 block	1	1	1	1	1	1
Allocation concealed	1	0	0	0	1	0	1	0
Baseline similarity	1	1	1	1	1	1	1	1
Patient blinding	0	1	1	0	1	0	0	0
Therapist blinding	0	1	1	0	0	0	0	0
Assessor blinding	1	1	1	1	0	1	1	1
<15 % drop outs	1	1	1	0	1	0	1	0
ITT analysis	1	0	0	1	1	0	1	0
Between group comparison reported	1	1	1	1	1	1	1	1
Post intervention point & variability measures	1	1	1	1	1	1	1	1
Total	8/10	8/10	8/10	6/10	8/10	5/10	8/10	5/10

#### Participants

There were 248 patients (ranged from 9 to 70 patients per study) in the experimental group and 235 patients (ranged from 9 to 72 patients per study) in the control group. Three of the trails [7, 25, 26] recruited community-dwelling participants, two trials recruited their outpatient study participants from medical educational and research centers [24, 28] and the other three trials [8, 27, 29] recruited their study participants from hospitals. The mean age range of the participants was 63.3 ± 8.0 to 75.7 ± 5.5 in the experimental group and 65.3 ± 8.5 to 75.7 ± 7.2 in the control group. In seven of the included articles, the disease severity of their study participants was recorded using the Hoehn and Yahr[H&Y] Scale and Patients with idiopathic PD with a baseline stage between 2 and 4 were recruited as a study participant [7, 8, 24, 25, 27–29].

#### Interventions

The experimental groups were treated with different treatment approaches. Five studies used postural adjustment and falls prevention strategies and balance training [7, 8, 25–27, 29],three studies used strengthening exercises [7, 26, 27], three studies applied gait training through overground walking and treadmill training [26, 28, 29] only one study [24] used PNF exercise and coordination training has been given for another one study [8].

Balance training was performed in the form of static, dynamic and functional balance training [7], in the form of exercises aimed at improving both feed forward and feedback postural reactions [8], in the form of highly challenging balance training (HiBT) that incorporates both dual-tasking and PD-specific balance components [25],in the form of stance- and gait tasks which require feedforward and feedback postural control [27] and in the form of technology-assisted balance training [29].

Strengthening exercises were performed with the aim to improve hip flexors, hip extensors and abductors, knee flexors and extensors, ankle dorsiflexors and plantar flexors [27], in the form of progressive lower limb strengthening [26], knee and hip extensors and hip abductors muscle strengthening [7].

Participants undertook training for 30 to 60 min per session for 7 to 24 weeks. Participants of the control group received no intervention in two studies [24, 28], visited by nurses [7], given joint mobilization and stretching exercises [8], asked to do physical activities [25], took medication and usual care [26] and provided strengthening exercise in two studies [27, 29].

#### Outcome measures

Three trails used berg’s balance scale of 0–56 scale range to measure the effect of training on balance outcome [7, 8, 28]. Three trials [25, 26, 28] used falls efficacy scale to assess balance and risk of falling. Falls risk in one study [26], UPDRS [27, 28], Fullerton Advanced balance scale [8, 27] and falls rate [8, 29] were also used as outcome measures to assess the level of balance dysfunction, postural instability, and risk of falling among patients with Parkinson’s disease (See Table 4).

**Table 4:** Summary of results of included randomized controlled trials (*n* = 8)

Reference	Results
(Ashburn et al. 2007) [7]	1. Functional reach test(cm): − Experimental group at (start/8 weeks/6 months) = 23.2/23.6/23.8
	Control group at (start/8 weeks/6months) = 25.0/24.0/22.5
	2. Berg balance scale(BBS) (o-56) : the higher the score, the risk of falling decreases
	Experimental group at (start/8 weeks/6 months) = 44.3/45.8/45.3
	Control group at (start/8weeks/6months) = 43.6./45.2/44.6
(Smania et al. 2010) [8]	1. BBS(0–56):- Experimental group (before/after/1 month) =44.5/49.8/49.9
	Control group (before/after/1 month) = 41.8/41.0/40.85
	2. Activities-Specific Balance Confidence Scale ABC(0–100):- Experimental group (before/after/1 month) = 54.3/61.3./62.3
	Control group (before/after/1 month) = 49.5/48.2/47.0
	3. Number of falls : Experimental group (before/after/1 month) = 4.3/1.3/1.3
	Control group (before/after/1 month) = 4.6/4.1/4.1
(Protas et al. 2005) [24]	Gait and step perturbation training resulted in a reduction in falls and improvements in gait and dynamic balance.
(Schlenstedt et al. 2015) [27]	1. FAB scale :- resistance group 22.2 ± 4.8
	Balance group 24.5 ± 4.6,(*P* value = 0.123)
(Conradsson et al. 2015) [25]	1. Falls Efficacy scale score:- Experimental group (baseline/post test = 30.1(/27.3
	Control group (baseline/post test = 28.8/26.5
(Shen and Mak 2014) [29]	There were fewer fallers in the expt. than in the Cont. group at Post 3 m, Post 6 m, and Post 12 m (P < .05). In addition, the expt. group had lower fall rate than the Cont. group at Post 3 m, 6 m and 15 m
(Allen et al. 2010) [26]	1. PD falls risk score: Experimental group (baseline(SD)/post test(SD) =34(25)/23(22)
	Control group (baseline(SD)/post test(SD) = 39(34)/38(31)
	2. Falls Efficacy scale score :- Experimental groups(baseline/post test = 28.1(12.1)/25.8(7.9)
	Control groups baseline/post test =29.1(10.3)/30.4(10.8)
(Cakit et al. 2007) [28]	1. BBS : Experimental group (baseline/8 weeks = 37.0 ± 9.41/44.09 ± 7.11
	Control Group (baseline/8 weeks = 42.6 ± 9.37/41.4 ± 10.65
	2. Falls Efficacy Scale : expt. group(baseline/8 weeks. = 37.72 ± 9.29/25.45 ± 7.46
	Control group(baseline/8 weeks. = 26.8 ± 8.06/29.2 ± 9.87

### Qualitative analysis of the effect of physiotherapy interventions on different outcomes

The effects of postural adjustment, fall prevention strategies, and balance training exercises on near falls and quality of life have been done by a study done in Southampton. The results showed that there was a tendency towards a reduction in fall events and injurious falls [7].

An RCT conducted in Italy brought that balance training showed significant improvements in declining PI and improving balance in patients with PD [8].

Another study conducted in the USA showed that Gait and step perturbation training can result in a reduction in falls and improvements in gait and dynamic balance for patients with PD [24].

According to a RCT conducted in Sweden, a HiBT regimen that incorporated both dual-tasking and PD-specific balance components (walking tasks on varying surfaces with or without visual constraints and voluntary arm/leg/trunk movements) significantly benefited balance and gait abilities when compared with usual care and showed promising transfer effects to everyday living [25].

Another comparative RCT done in Germany found that it is effective to use both coordinated resistance and balance training to improve balance and postural control for patients with PD [27].

A study done in china on the effectiveness of technology-Assisted Balance and Gait training found that the balance and gait training program assisted by technological devices reduced the number of fallers and the fall rate compared with the strength training program. It supported the clinical use of balance and gait training for reducing fall events in people with PD [29].

The effects of an exercise program on reduction of fall risk factors in People with PD were determined by a study done in Australia. It found that there were trends towards improvement in the exercise group for measures of muscle strength, walking, and fear of falling, but there was a lack of improvement in balance outcomes [26].

A study done in turkey on the effects of incremental speed-dependent treadmill training on postural instability and fear of falling found that specific exercise programs using incremental speed-dependent treadmill training may improve mobility, reduce postural instability and fear of falling in patients with Parkinson’s disease [28].

The effects of physiotherapy interventions on different outcome measures are summarized in Table 4.

### Meta- analysis on effects of physiotherapy interventions on berg balance scale

**Fig. 2:**
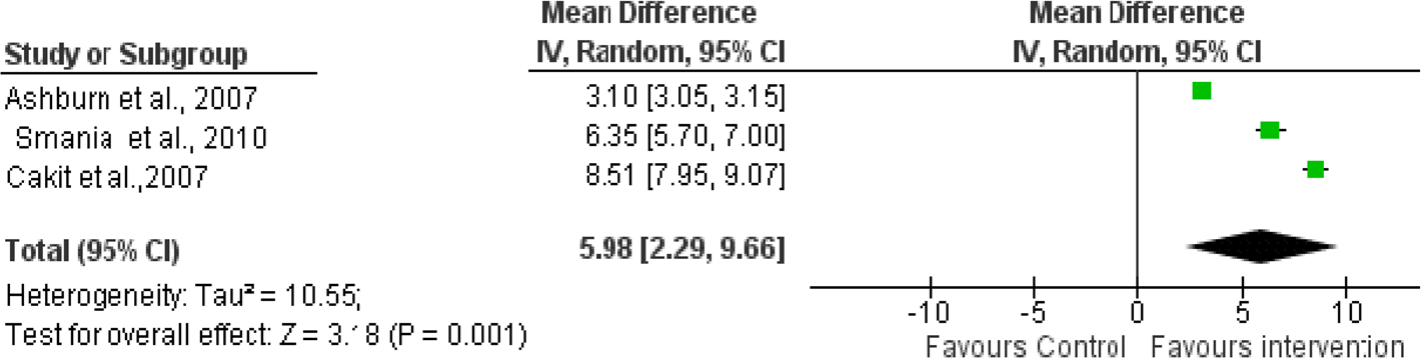
Comparison of physiotherapy interventions with controls in relation to the Berg balance scale

The effects of muscle strengthening, range of movement, balance training, walking training, Exercises of self-destabilization of the center of body mass and incremental speed-dependent treadmill training on berg balance scale(BBS)immediately after intervention period was examined by pooling data from three trials involving 239 participants. The pooled data indicated that physiotherapy exercises increased BBS by 5.98 (95 % CI-2.29 to 9.66,*p* = 0.001) than the control group (Fig. 2).

### Meta- analysis on effects of physiotherapy interventions on falls efficacy scale

**Fig. 3:**
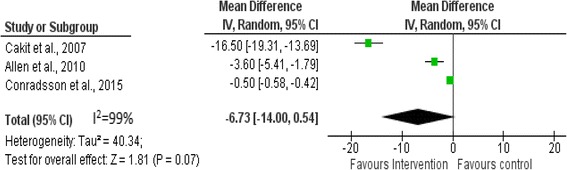
Comparison of physiotherapy interventions with controls in relation to falls efficacy scale

The effects of muscle strength, balance training, freezing and reactive postural adjustments in controlling balance during single-tasking compared with normal physical activities and participation in ongoing rehabilitation program was examined by pooling data from three studies involving 167participants. The pooled data indicated that these physiotherapy exercises decreased the incidence of falling by 6.73: (95 % CI: −14.00, 0.54, *p* = 0.07) with the overall effect of *Z* = 1.81. However, it was not significant. There was heterogeneity between the studies (*I*^2^ = 99 %) (See Fig. 3).

## Discussion

The objective of this systematic review was to evaluate the current evidence for benefits of physiotherapy treatments for treating balance impairment, postural instability and reducing the tendency and frequency of falling for patients with idiopathic Parkinson’s disease.

The overall result of this systematic review of RCTs indicates that multifactorial physiotherapy interventions like muscle strengthening, range of movement, balance training and walking training exercises were found to have a positive effect on treating BD and PI among idiopathic patients with PD. But the effect of training intensity, duration, and modality is variable and inconsistent.

In this systematic review, different balance training techniques were found to be effective in improving balance and they were administered in the form of static, dynamic and functional training [7]. Exercises aimed at improving both feed forward and feedback postural reactions [8], HiBT that incorporates both dual-tasking and PD-specific balance components [25], stance- and gait tasks which require feed forward and feedback postural control [27] and technology assisted balance training exercises [29] also demonstrated a very promising outcome of balance improvement. This finding is supported by a meta-analysis which found that exercises and motor training can improve the performance of balance-related activities in people with PD [12].

Physiotherapy interventions targeted at preventing falls and Exercises of self-destabilization of the Center of body mass during walking and locomotor dexterity have an impact on reinforcing the need to focus attention on maintaining balance when performing mobility tasks in a standing position [7, 8]. This result was found by two studies which have the following limitations: Increasing numbers of control subjects who accessed rehabilitation outside of the trial by 6 months [7], lack of a follow-up assessment at 3 or more months after training and lack of assessment of some important parameters related to balance and PI [8].

This systematic review showed that repetitive exercises, HiBT, and incremental speed-dependent treadmill training will help to improve range of motion, endurance, gait parameters, functional reaching activities and postural stability in particular and balance at large. It also showed that those exercises help to decrease fall rate and fear of falling which could have the direct or indirect contribution in improving balance [7, 24–26, 28]. However, the results of a study done on the effects of HiBT [25] can only be generalized to elderly, specifically community-dwelling individuals with mild- to moderate-stage PD without known cognitive impairments.

Other limitations of these studies include a majority of the participants were recruited by advertisement, a method that can lead to a convenience sample of individuals interested in training and improving balance abilities [25], did not attempt to prevent participants from changing their medications during the study period for ethical reasons [26], relatively small sample size and unable to address the intensity, frequency, and duration of the training intervention [24] and having small sample size [28].

The difference between resistance and balance training to improve postural control and balance in people with PD have also been analyzed in this systematic review and weak evidence was found that freely coordinated resistance training might be more effective than balance training [27]. Nevertheless, the major limitation of this RCT is that training frequency was low and probably under-dosed to detect significant differences between these two competing training types. Second, it had a 20 % drop-out rate which might have been underpowered to detect significant differences. Furthermore, they did not assess fall rates which would be of interest as strength and balance performance are independent risk factors for falls. Finally, they did not include any control group without any intervention which would allow to further interpret the effects of both training types [27].

Technology assisted balance and gait training have been found significant in reducing the number of fallers at Post 3 month, 6 months, and 12 months. In addition, it also showed that a lower fall rate than the Control group was registered [29]. However, the included study has several limitations. First, the sample size and statistical power were not adequate to detect group differences. Second, there was a possible placebo effect since subjects were not blinded to group assignment. Third, all of the subjects were community-dwelling people with a mild to moderate disease level. Fourth, they used monthly phone follow-up registration of fall incidence instead of using a fall diary because most of the subjects did not have education beyond the elementary level and some were even illiterate. Fifth, the dropout rate of 31 % was relatively high. Therefore, the results cannot be generalized to patients with advanced-stage PD or those who have been institutionalized and educated [29].

This meta-analysis indicated that a significant difference was obtained on physiotherapy intervention for improving balance. However, there was not a significant difference was obtained on physiotherapy intervention for improving postural stability.

A meta-analysis of the effects of exercise and motor training on balance and falls in PD supported our finding. It concluded that there was a significant but small benefit of physiotherapy interventions on balance-related performance measures. However, there was no beneficial effect on falls in PD [30].

### Limitation of this systematic review

Addressing all important outcome measures was not possible. No attempts were made to source unpublished studies, nor studies published in languages other than English. The authors suggestively agreed that unpublished trials may have poor methodology over the published ones. The review had feasibility constraint over translation for other language trails.

## Conclusion

The results of this systematic review with meta-analysis concluded that physiotherapy interventions like balance training combined with muscle strengthening, the range of movement, walking training exercise is effective in improving balance in patients with PD and more effective than balance exercises alone.

HiBT and incremental speed-dependent treadmill training can also be part of a rehabilitation program for management of balance and Postural instability in patients with idiopathic PD.

### Clinical application

This review suggests that physiotherapy techniques, exercises, and balance training appear to result in comparable outcomes for balance, postural stability, and reduction in falls. Consequently, prescription of balance and walking training exercise, repetitive exercises, HiBT and incremental speed-dependent treadmill training for idiopathic PD may pledge substantial improvement. Therefore, balance training exercises should be incorporated into a plan of care in conjunction with other necessary interventions to make the patient independent as much as possible.

## Abbreviations

ABC, activities-specific balance confidence scale; BBS, berg balance scale; BD, balance dysfunction; BEST, balance evaluation systems test; COBM, center of body mass; FAB, Fullerton advanced balance scale; FES, falls efficacy scale; H&Y, Hoehn and Yahr; HiBT, highly challenging balance training; *P* value (Z), *P* value and corresponding *Z* value; PD, Parkinson’s disease; PI, postural instability; QOL, quality of life in persons; RCT, randomized controlled trial; UPDRS, unified Parkinson’s disease rating scale
